# Environmental Effects on Vertebrate Species Richness: Testing the Energy, Environmental Stability and Habitat Heterogeneity Hypotheses

**DOI:** 10.1371/journal.pone.0035514

**Published:** 2012-04-18

**Authors:** Zhenhua Luo, Songhua Tang, Chunwang Li, Hongxia Fang, Huijian Hu, Ji Yang, Jingjing Ding, Zhigang Jiang

**Affiliations:** 1 Key Laboratory of Animal Ecology and Conservation Biology, Institute of Zoology, Chinese Academy of Sciences, Beijing, China; 2 Graduate School of Chinese Academy of Sciences, Beijing, China; 3 South China Institute of Endangered Animals, Guangdong Academy of Sciences, Guangzhou, China; 4 Jiangsu Academy of Forestry, Nanjing, China; Utah State University, United States of America

## Abstract

**Background:**

Explaining species richness patterns is a central issue in biogeography and macroecology. Several hypotheses have been proposed to explain the mechanisms driving biodiversity patterns, but the causes of species richness gradients remain unclear. In this study, we aimed to explain the impacts of energy, environmental stability, and habitat heterogeneity factors on variation of vertebrate species richness (VSR), based on the VSR pattern in China, so as to test the energy hypothesis, the environmental stability hypothesis, and the habitat heterogeneity hypothesis.

**Methodology/Principal Findings:**

A dataset was compiled containing the distributions of 2,665 vertebrate species and eleven ecogeographic predictive variables in China. We grouped these variables into categories of energy, environmental stability, and habitat heterogeneity and transformed the data into 100×100 km quadrat systems. To test the three hypotheses, AIC-based model selection was carried out between VSR and the variables in each group and correlation analyses were conducted. There was a decreasing VSR gradient from the southeast to the northwest of China. Our results showed that energy explained 67.6% of the VSR variation, with the annual mean temperature as the main factor, which was followed by annual precipitation and NDVI. Environmental stability factors explained 69.1% of the VSR variation and both temperature annual range and precipitation seasonality had important contributions. By contrast, habitat heterogeneity variables explained only 26.3% of the VSR variation. Significantly positive correlations were detected among VSR, annual mean temperature, annual precipitation, and NDVI, whereas the relationship of VSR and temperature annual range was strongly negative. In addition, other variables showed moderate or ambiguous relations to VSR.

**Conclusions/Significance:**

The energy hypothesis and the environmental stability hypothesis were supported, whereas little support was found for the habitat heterogeneity hypothesis.

## Introduction

The variability of spatial patterns of species richness and its underlying mechanisms at large scales are hot debates in macroecology and biogeography [1]–[3]. Research on plants, invertebrates, fish, amphibians, reptiles, birds and mammals has been conducted at global, regional, and local scales, to document species richness patterns and explore the impacts of biotic and abiotic biogeographical factors [4]–[10], such as the environment and habitat [11]–[16]. These effects are obvious, but intense debates still exist regarding the underpinning mechanism, while comprehensive explanations of the source of species richness variation remain controversial [17], [18]. Therefore, more detailed studies are needed to support these arguments [19], [20]. Over the years, more than 30 competing hypotheses have been proposed [1], [18]–[22]. Among these hypotheses, the energy hypothesis, the environmental stability hypothesis and the habitat heterogeneity hypothesis are the most frequently mentioned. Several investigations have provided evidence supporting those hypotheses, but a clear cause-effect relationship has not yet been found [6].

The energy hypothesis posits that higher productivity, ambient energy and water-energy dynamics result in higher species diversity [16], [23]–[26]. Areas with higher solar radiation and precipitation have higher primary production and they promote thermoregulation, growth, reproduction, differentiation, and the evolution of species, thus leading to higher biomass, larger population sizes, lower extinction rates, and ultimately more co-occurring species, i.e., higher biodiversity [26]–[29]. The environmental stability hypothesis proposes that species are expected to have broader environmental tolerances if they are to survive with greater environmental variation, which will lead to an extension of the range for each species, a reduction of the number of co-existing species in an area, i.e., a decrease in species richness. In contrast, a stable environment could accelerate species specialization and ecological niche diversification, which will increase the environmental capacity for species richness [30]–[33]. The habitat heterogeneity hypothesis claims that topographical and spatial variation, such as elevation range, landscape, or vegetation variability, could produce mosaics and gradients for critical resources affecting co-existing species, thereby leading to higher biodiversity [34]–[36].

Ideally, studies of species richness patterns should encompass large areas at a macro-scale, because misleading results may occur if studies are performed under conditions of partial coverage [6]. With its vast territory, wide latitudinal range, complex terrain and diverse climate, China is one of the top twelve mega-biodiversity countries in the world [37]. Furthermore, biodiversity surveys have been conducted nation-wide in China over recent decades. This offers a perfect opportunity to study the impacts of biogeographical factors on species richness gradient. However, examination of patterns of total vertebrate species richness in this region has been limited [38], [39]. In this study, we compiled the distributions of vertebrate species and produced a dataset of predictive variables to: (1) examine the relationships among vertebrate species richness (VSR) and the factors of energy, environment stability, and habitat heterogeneity; and (2) test the energy hypothesis, the environment stability hypothesis, and the habitat heterogeneity hypothesis with VSR.

## Methods

**Figure 1:**
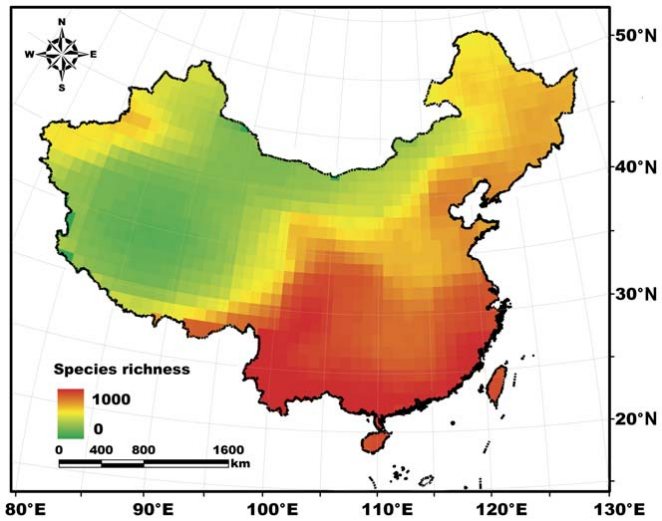
Vertebrate species richness pattern of China at the scale of 100×100 km. The species richness was calculated by overlaying the distributions of mammals, birds, reptiles and amphibians. The colour gradient represented vertebrate species richness.

This study was conducted across the mainland and the two main islands (Taiwan and Hainan) of China, at latitudes ranging from 18°N to 54°N and longitudes ranging from 73°E to 135°E (Figure 1).

### Data organization

We compiled an exhaustive database containing the distributions of 2665 vertebrate species, including 625 mammals, 1330 birds, 402 reptiles, and 298 amphibians, based on Fei (1999), MacKinnon *et al*. (2000), Ji and Wen (2002), Baillie *et al*. (2004), Sheng *et al*. (2005), Pan *et al*. (2007) and the Vertebrate Species Information Database of our own research group [40]–[46]. We excluded marine and aquatic species, whose geographical ranges are distinct from terrestrial animals. Any species that were subject to taxonomic disputes or that lacked comprehensive distributional information were also removed from the overall data set. As a result, a total of 365 species (110 mammals, 135 birds, 54 reptiles, and 66 amphibians) were excluded, leaving 2290 terrestrial species for the analysis. We digitized the range maps at a scale of 1×1 km and updated them by adding new distribution records (recorded after the original publications) of these species, which were collected from comprehensive published papers, faunistic atlases, nature reserve biodiversity survey reports, documents of museum collections, and field survey records from our laboratory over the past seventeen years [47], [48]. We then overlaid all the range maps to calculate the VSR for each grid cell.

We used eleven ecogeographic variables (all at a scale of 1×1 km), which were classified into three categories: (i) Energy: annual mean temperature (ANMT, °C), annual precipitation (ANPR, mm), and normalized difference vegetation index (NDVI); (ii) environment stability: temperature seasonality (TS, °C), temperature annual range (TEMR, °C), and precipitation seasonality (PRS, mm); and (iii) habitat heterogeneity: altitude range (ATR, m), slope (SLP, °), aspect (ASP), land cover diversity (Landcover), and vegetation type diversity (Vegetation type). ANMT, TS, TEMR, ANPR, and PRS were obtained from WorldClim 1.4 at http://www.worldclim.org/ [49]. ATR, SLP, and ASP were calculated using a 1-km^2^ digital elevation model (DEM) obtained from http://srtm.csi.cgiar.org/ [12], [50]. Land cover data was derived from Global Landcover 2000 at http://ies.jrc.ec.europa.eu/global-land-cover-2000 [51]. We calculated the annual mean NDVI in 1998 by averaging the monthly NDVI layers from http://www.data.ac.cn/ [52]. The vegetation type data was obtained from the China Vegetation Database [53].

Analyses based on range map data represent species coexistence and distributions at some relatively coarse scales, because species do not occur everywhere within their geographical ranges [54]–[56]. Thus, our 1×1 km range map based VSR values may be not actually realistic. To overcome this problem, we re-sampled the VSR and all the rasters of predictive variables into a 100×100 km resolution, following Ding *et al*. (2006) [6]. We counted the numbers of species, land cover, and vegetation types in each 100×100 km grid cell and used them as the variables of vertebrate species richness (VSR), land cover diversity (Landcover), and vegetation type diversity (Vegetation type). As to ATR, we extracted the difference between the maximum and minimum altitudes in each 100×100 km grid cell. We conducted the mean altitude of each 100×100 km grid cell based on the 1-km^2^ DEM, and calculated ASP and SLP for each grid. ASP was classified according to (class: label (value range)): North: 1 (337.5° (−22.5°)–22.5°), Northeast: 2 (22.5°–67.5°), East: 3 (67.5°–112.5°), Southeast: 4 (112.5°–157.5°), South: 5 (157.5°–202.5°), Southwest: 6 (202.5°–247.5°), West: 7 (247.5°–292.5°) and Northeast: 8 (292.5°–337.5°(−22.5°)) (0° was defined as the direction of North). The rest of the variables and the VSR were re-sampled by averaging procedures for each of the 100×100 km grid cell.

### Statistical analyses

To test the energy hypothesis, the environmental stability hypothesis, and the habitat heterogeneity hypothesis, we generated the best-fit predictive models between the VSR and each of the three variable groups based on an information theoretic approach [57], [58]. For each variable group, we used generalized linear models (GLMs) to establish a set of candidate models that including all the possible combinations of variables, and used Akaike's information criterion (AIC) to compare these candidate models by ranking them with *ΔAICc* [57]. We chose the model with *ΔAICc* = 0 as the best-fit model and the relative likelihood of each candidate model was assessed by Akaike weight (*AICw*) [57]. We calculated the *R* ^2^ value of the GLM to assess the explanatory power of the best-fit model to the VSR. In order to evaluate the relative importance of the predictive variables in each group, we followed Burnham and Anderson (2002) to sum the *AICw*s over all models that included a given variable [57], [58]. Furthermore, correlation analyses were performed to identify the relationships of the predictive variables in the best-fit models and the VSR. We used linear and second-order polynomial models and we selected the model with higher explanatory power (*R* ^2^) for each variable.

To determine if spatial autocorrelation was important for our models, we followed the method of Diniz-Filho *et al*. (2003) to calculate the Moran's *I* coefficients for the VSR and the residuals of the best-fit GLMs for variable groups at ten distance classes (100, 200, 300, 400, 500, 600, 700, 800, 900, and 1000 km) in SAM 4.0 [59]. Our statistical analyses were performed using SPSS Version 13.0 and SAS Version 9.1. All the analyses were considered significant at *P*<0.05. The spatial analyses were conducted with ESRI ArcGIS 9.2, and all coordinates were transferred into WGS 1984 UTM Zone 50N.

## Results

### Spatial pattern of vertebrate species richness

At the scale of 100×100 km grid size, the VSRs were 137 to 956 with an average of 398.2±140.1 (mean ± SD)(Figure 1). Generally, the VSR decreased from southeast to northwest in China (Figure 1). Only 12.9% of the 100×100 km grids contained more than 500 species. These high VSR grids were mainly located in the southwestern areas, tropics, and sub-tropics of the country, which contained several hot-spots, including the Hengduan Mountains, the Xishuangbanna region of Yunnan Province, the southeastern and southern coasts, Hainan, and Taiwan (Figure 1). The grids containing 200–500 species were mainly concentrated in the vast eastern and northeastern plains of the country, which accounted for 49.2% of the total of grid cells. The remaining grid cells (37.9% of the total) had VSRs of <200 and they were mainly located in the northwestern areas and Qinghai-Tibetan Plateau (Figure 1).

### Spatial autocorrelation

**Figure 2:**
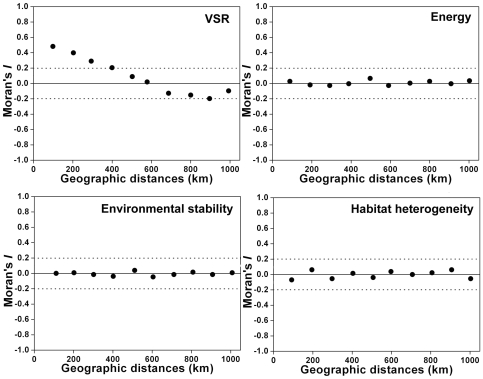
Spatial correlograms (Moran's *I* coefficients) for the VSR and the residuals of the best-fit GLMs of energy, environmental stability, and habitat heterogeneity variable groups. Ten distance classes (100, 200, 300, 400, 500, 600, 700, 800, 900, and 1000 km) were included. The dotted lines represented the Moran's *I* values of 0.2 and −0.2.

The spatial correlogram for the VSR showed a strong spatial structure, as decreasing highly positive autocorrelation coefficients were detected up to 400 km (Moran's *I*>0.2) (Figure 2). At the distance classes from 500 to 1000 km, the Moran's *I* coefficients were between −0.2 and 0.2 (Figure 2). The spatial correlograms for the residuals of the best-fit models of energy, environmental stability, and habitat heterogeneity variable groups showed that their Moran's *I* values were all close to zero (Figure 2). We considered that our models successfully eliminated most spatial autocorrelation in the species richness data.

### Testing the species richness hypotheses

The results of AIC-based model selection showed that the best-fit model between VSR and energy variables included all the predictive factors in this group, which explained 67.6% of VSR variation (*AICw* = 0.636, *F* = 221.439, *P*<0.001, *R* ^2^ = 0.676; Table 1). ANMT was the most significant variable for the VSR, with a sum of *AICw*s of 0.950 (Table 2). The next most important variable was ANPR, with a sum of *AICw*s equal to 0.887, followed by NDVI (sum of *AICw*s = 0.799) (Table 2). The best-fit model for environmental stability factors included two variables (TEMR and PRS) and explained 69.1% of the variation of VSR (*AICw* = 0.685, *F* = 344.429, *P*<0.0001, *R* ^2^ = 0.691; Table 1), where TEMR and PRS had summed *AICw*s of 0.989 and 0.961 respectively, irrespective of the effect of TS (sum of *AICw*s = 0.315) (Table 2). ATR (sum of *AICw*s = 0.821), Vegetation type (sum of *AICw*s = 0.729), and ASP (sum of *AICw*s = 0.723) were included in the best-fit model of habitat heterogeneity variables (Table 2), which explained only 26.3% of the variation of VSR (*AICw* = 0.789, *F* = 42.512, *P* = 0.017, *R* ^2^ = 0.246; Table 1). In addition, SLP and Landcover were excluded with *AICw* sums of 0.431 and 0.279 (Table 2).

**Table 1:** The best-fit models among the VSR and energy, environmental stability, and habitat heterogeneity variables, based on AIC-based model selection.

Models	Δ*AICc*	*AICw*	*F*	*P*	*R^2^*
**Energy hypothesis**					
ANMT+ANPR+NDVI	0.000	0.636	221.439	<0.001	0.676
**Environmental stability hypothesis**					
TEMR+PRS	0.000	0.685	344.429	<0.0001	0.691
**Habitat heterogeneity hypothesis**					
ATR+ASP+Vegetation type	0.000	0.489	42.512	0.017	0.263

**Table 2:** Relative importance of the predictive variables in energy, environmental stability, and habitat heterogeneity variable groups.

Variable	Sum of *AICw*s
**Energy hypothesis**	
ANMT	0.950
ANPR	0.887
NDVI	0.799
**Environmental stability hypothesis**	
TEMR	0.989
PRS	0.961
TS	0.315
**Habitat heterogeneity hypothesis**	
ATR	0.821
Vegetation type	0.792
ASP	0.723
SLP	0.431
Landcover	0.279

The relative importance of a given variable was explained by the sum of *AICw*s over all candidate models that include this variable.

**Figure 3:**
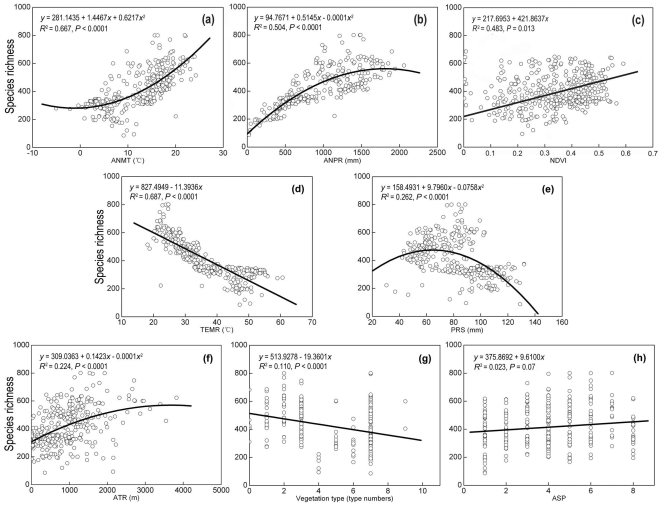
Relationships between vertebrate species richness and the variables included in the best-fit model that testing the three species richness hypotheses. Linear and second-order polynomial models were used and only the models with higher explanatory powers (*R* ^2^) were showed here. ASP was defined as (class: label (value range)) North: 1 (337.5° (−22.5°) −22.5°), Northeast: 2 (22.5°–67.5°), East: 3 (67.5°–112.5°), Southeast: 4 (112.5°–157.5°), South: 5 (157.5°–202.5°), Southwest: 6 (202.5°–247.5°), West: 7 (247.5°–292.5°) and Northeast: 8 (292.5°–337.5°(−22.5°)) (0° was defined as the direction of north).

Correlation analyses indicated that VSR was strongly, positively, and nonlinearly related to ANMT (*P*<0.0001, *R* ^2^ = 0.667; Figure 3(a)) and ANPR (*P*<0.0001, *R* ^2^ = 0.504; Figure 3(b)). A significant, positive, and linear correlation was detected between vertebrate species richness and NDVI (*P* = 0.013, *R* ^2^ = 0.483; Figure 3(c)). The relationship between VSR and TMPR was strong and linear, with species number decreasing as TMPR increased (*P*<0.0001, *R* ^2^ = 0.687; Figure 3(d)). VSR had a moderate and nonlinear relationship with PRS (*P*<0.0001, *R* ^2^ = 0.262; Figure 3(e)), while ATR (*P*<0.0001, *R* ^2^ = 0.224; Figure 3(f)) and Vegetation type (*P*<0.0001, *R* ^2^ = 0.110; Figure 3(g)) were positively and negatively related to VSR, respectively. In addition, ASP (*P* = 0.07, *R* ^2^ = 0.023; Figure 3(h)) explained limited variation and it was not significantly associated with vertebrate species richness (*R* ^2^<0.1).

## Discussion

### The energy hypothesis

Energy is essential for the survival of animals, and the dynamics of its availability may induce changes in the species richness gradient compared with their initial condition [26], [60]. The energy supply also determines the environmental capacity of species diversity [61]. Thus, higher energy levels support more species, because it maintains more individuals of each species and avoids extinction [6], [24], [25]. In related studies of plants and animals, the energy hypothesis was considered the critical mechanism for the species richness spatial pattern [26], [62]–[67]. If this hypothesis is true, the species richness should positively and monotonically correlate with the mean conditions of temperature, precipitation or primary productivity [6].

This study supported the energy hypothesis, because all three energy-related factors (ANPR, ANMT, and NDVI) had high values of relative importance and, when combined these variables, explained more than 60% of VSR variation. The robust, positive relationships between ANPR, ANMT, and NDVI with VSR indicated that higher ambient energy and a greater water supply could sustain more species. VSR decreased with increasing latitude in China, and the highest VSR was located between 20°N to 30°N, which had the highest temperature and precipitation. Similar conclusions were reached in the analyses of vertebrates in the whole Americas [26], [29]. Research on butterflies, birds, reptiles, and plants has also supported this hypothesis [68]–[71]. NDVI is considered as an index of primary productivity in ecosystem, and higher NDVI implies higher energy input. Our results showed a significant trend of increase in vertebrate species number with increasing NDVI, which indicates low latitudinal areas, particularly tropical regions, support higher VSR. Ding *et al*. (2006) reported a similarly positive relationship between bird species diversity and NDVI in East Asia, especially on the mainland, and regarded energy (primary productivity) as the best factor in explaining species richness. This suggests that the energy hypothesis may play an important role in patterns of biodiversity [6].

### The environmental stability hypothesis

The environmental stability hypothesis suggests that stable environmental conditions can increase species diversity, because they narrow the niche widths, increase the number of ecological niches, and promote specialization in species [31], [32]. If this hypothesis holds true, areas with less variation in climate (e.g. low latitudes rather than high latitude areas) should contain larger numbers of species. Our results supported this hypothesis, because TEMR and PRS accounted for nearly 70% of the VSR variation and both variables showed >0.95 relative importance. The strongly negative relationship of TEMR and species richness indicated that lower temperature variation could allow more species to co-exist and it significantly increases species diversity in an area. Meanwhile, a general decreasing trend of VSR was emerged with rising PRS, although their correlation was moderate and nonlinear. Thus, the hot and wet tropics and sub-tropics in China have lower temperature and precipitation variation and they contain more vertebrate species. This hypothesis was also supported by Lin *et al*. (2009), who concluded that TEMR was one of the main contributory factors to mammalian biodiversity in China [20]. Similar results were also reported by Qian *et al*. (2009), Altamirano *et al*. (2010), and Carrara and Vazquez (2010) for birds and mammals in the Americas and woody plants in temperate Andean forests [26], [72], [73].

### The habitat heterogeneity hypothesis

The habitat heterogeneity hypothesis suggests that higher diversity in topographical and spatial habitat structures could permit finer subdivisions of the limiting resources, produce diverse and sufficient ecological niches, promote greater specialization and greater co-existence of species, thereby increasing species richness and community composition [34], [35]. Topographic heterogeneity was found to account for a high proportion of the species richness pattern at the macro-scale [6], [72], [73]. Spatial heterogeneity was shown to have an important role in shaping the species richness gradient at finer-scales in previous studies of birds and mammals [17], [35], [74]–[77], which could be considered as variations of landscape and vegetation [16], [24], [36], [62], [78].

In our results, the predictive variables for habitat heterogeneity (ATR, Vegetation type, and ASP) explained only one fourth of the VSR variation, where ATR and Vegetation type had subequal importance and followed by SLP. The results indicated that higher altitude ranges could moderately increase species richness, whereas Vegetation type and SLP had ambiguous or even slightly negative correlations with VSR. This was possibly a consequence of the scale-dependent effects of habitat heterogeneity [73], [77]. Topography heterogeneity might generate a specific effect on biodiversity at the national scale, but the influence of spatial heterogeneity was secondary. Similarly, Lin *et al*. (2009) took the number of ecosystems in an area as a parameter of habitat heterogeneity and concluded that it was a key factor affecting mammalian biodiversity variation in China [20].

### Conclusion

The species richness of vertebrates shows a decreasing pattern from the southeast to the northwest in China. Several regions in the southwestern mountainous areas, tropics/sub-tropics along the southeastern/southern coasts, Taiwan, and Hainan are with abundant vertebrate species and should be received more attention in the national biodiversity conservation system. The spatial gradient of vertebrate species richness corresponds to energy and environmental stability gradients significantly, in which mean conditions and variations of temperature, precipitation, and NDVI play important roles. The energy hypothesis and the environment stability hypothesis are supported by the data from this country, as vertebrate diversity increases with the increases of annual mean temperature, precipitation, and NDVI, whereas decreases with the increases of variations in temperature and precipitation. By contrast, the habitat heterogeneity hypothesis is not well supported, as topographic, land cover, and vegetation heterogeneities only account for limited variation in species richness in this area, and barely positive correlations or even ambiguous relationships are detected between biodiversity and these habitat heterogeneity factors. In general, our study provides a brief analysis on biodiversity gradient and its mechanisms in China, and could produce a basic reference in establishing of biodiversity conservation strategies and nature reserves.

## References

[1] Brown JH, Lomolino MV (1998). Biogeography, 2nd edn.

[2] Gaston KJ, Blackburn TM (2000). Pattern and process in macroecology.

[3] Hillebrand H (2004). On the generality of the latitudinal diversity gradient.. The American Naturalist.

[4] Francis AP, Currie DJ (2003). A globally consistent richness-climate relationship for angiosperms.. The American Naturalist.

[5] Rodríguez MA, Belmontes JA, Hawkins BA (2005). Energy, water and large-scale patterns of reptile and amphibian species richness in Europe.. Acta Oecologica.

[6] Ding T, Yuan H, Geng S, Koh C, Lee P (2006). Macro-scale bird species richness patterns of the East Asian mainland and islands: energy, area and isolation.. Journal of Biogeography.

[7] White P, Kerr JT (2006). Contrasting spatial and temporal global change impacts on butterfly species richness during the 20th century.. Ecography.

[8] Zhao S, Fang J, Peng C, Tang Z (2006). The relationships between terrestrial vertebrate species richness in China's nature reserves and environmental variables.. Canadian Journal of Zoology.

[9] Qian H, Ricklefs RE (2007). A latitudinal gradient in large-scale beta diversity for vascular plants in North America.. Ecology Letters.

[10] Huntley B, Collingham YC, Willis SG, Green RE (2008). Potential impacts of climatic change on European breeding birds.. PLoS ONE.

[11] Rahbek C, Graves GR (2001). Multiscale assessment of patterns of avian species richness.. Proceedings of the National Academy of Sciences USA.

[12] Hortal J, Rodríguez J, Nieto-Díaz M, Lobo JM (2008). Regional and environmental effects on the species richness of mammal assemblages.. Journal of Biogeography.

[13] Mellin C, Bradshaw CJA, Meekan MG, Caley MJ (2010). Environmental and spatial predictors of species richness and abundance in coral reef fishes.. Global Ecology and Biogeography.

[14] Roy K, Jablonski D, Valentine JW, Rosenberg G (1998). Marine latitudinal diversity gradients: tests of causal hypotheses.. Proceedings of the National Academy of Sciences USA.

[15] Sala OE, Chapin FS, Armesto JJ, Berlow E, Bloomfield J (2000). Biodiversity-global biodiversity scenarios for the year 2100.. Science.

[16] Connell JH, Orias E (1964). The ecological regulation of species diversity.. The American Naturalist.

[17] MacArthur RH, Connell JH (1966). The biology of populations.

[18] Willig MR, Kaufman DM, Stevens RD (2003). Latitudinal gradients of biodiversity: pattern, process, scale, and synthesis.. Annual Review of Ecology, Evolution and Systematics.

[19] Rosenzweig ML (1995). Species diversity in space and time.

[20] Lin X, Wang Z, Tang Z, Zhao S, Fang J (2009). Geographic patterns and environmental correlates of terrestrial mammal species richness in China.. Biodiversity Science.

[21] Rohde K (1992). Latitudinal gradients in species diversity: the search for the primary cause.. Oikos.

[22] Whittaker RJ, Willis KJ, Field R (2001). Scale and species richness: towards a general hierarchical theory of species diversity.. Journal of Biogeography.

[23] Wang Z, Tang Z, Fang J (2009). The species-energy hypothesis as a mechanism for species richness pattern.. Biodiversity Science.

[24] Wright DH (1983). Species-energy theory: an extension of species-area theory.. Oikos.

[25] Hawkins BA, Field R, Cornell HV, Currie DJ, Guégan JF (2003). Energy, water, and broad-scale geographic patterns of species richness.. Ecology.

[26] Carrara R, Vázquez DP (2010). The species-energy theory: a role for energy variability.. Ecography.

[27] Clarke A, Gaston KJ (2006). Climate, energy and diversity.. Proceedings of the Royal Society B: Biological Sciences.

[28] Turner JRG, Gatehouse CM, Corey CA (1987). Does solar energy control organic diversity? Butterflies, moths and the British climate.. Oikos.

[29] Currie DJ (1991). Energy and large-scale patterns of animaland plant-species richness.. The American Naturalist.

[30] O'Brien E (1993). Climatic gradients in woody plant species richness: towards an explanation based on an analysis of southern Africa's woody flora.. Journal of Biogeography.

[31] Klopfer PH (1959). Environmental determinants of faunal diversity.. The American Naturalist.

[32] Klopfer PH, MacArthur R (1960). Niche size and faunal diversity.. The American Naturalist.

[33] Stevens GC (1989). The latitudinal gradient in geographical range: how so many species coexist in the tropics.. The American Naturalist.

[34] Pianka ER (1966). Latitudinal gradients in species diversity: a review of concepts.. The American Naturalist.

[35] Kerr JT, Packer L (1997). Habitat heterogeneity as a determinant of mammal species richness in high energy regions.. Nature.

[36] Hugo S, van Rensburg BJ (2008). The maintenance of a positive spatial correlation between South African bird species richness and human population density.. Global Ecology and Biogeography.

[37] Chen C (1998). China's Biodiversity: A Country Study.

[38] Zhang R, Lin Y (1985). The distribution tendency of land mammals in China and adjacent areas.. Acta Zoologica Sinica.

[39] Zhao S, Fang J, Peng C, Tang Z, Piao S (2006). Patterns of fish species richness in China's lakes.. Global Ecology and Biogeography.

[40] Baillie JEM, Hilton-Taylor C, Stuart SN (2004). 2004 IUCN red list of theatened species: a global species assessment.. http://www.iucnredlist.org/technical-documents/spatial-data.

[41] Sheng H (2005). Atlas of mammals of China.

[42] Pan Q, Wang Y, Yan K (2007). A field guide to the mammals of China.

[43] MacKinnon J, Phillipps K, He F (2000). A field guide to the birds of China.

[44] Ji D, Wen S (2002). Atlas of reptiles of China.

[45] Fei L (1999). Atlas of amphibians of China.

[46] Jiang Z, Li Y, Li C, Fang H, Wu J, Yang C (2009). Species abundance in China, a test of climate hypothesis.. Modern ecology forum IV: theory and practice.

[47] Jiang Z, Tang S, Luo Z, Fang H, Li C (2012). Survey report on key wild terrestrial vertebrates in the carst region of southwest China.

[48] Jiang Z, Luo Z, Tang S, Fang H, Li C, Meng Z (2012). Assessment report on threatened status of terrestial vertebrates in China.

[49] Hijmans RJ, Cameron SE, Parra JL, Jones PG, Jarvis A (2005). Very high resolution interpolated climate surfaces for global land areas.. International Journal of Climatology.

[50] CGIAR International Research Centers (1999). The CGIAR consortium for spatial information (CGIAR-CSI).. http://srtm.csi.cgiar.org/.

[51] European Commission, Joint Research Centre, Institute for Environment and Sustainability (2003). Global Land Cover 2000 Database.. http://ies.jrc.ec.europa.eu/global-land-cover-2000/.

[52] Institute of Geographic Sciences and Natural Resources Research, Chinese Academy of Sciences (2002). Thematic Database of Human-earth System.. http://www.data.ac.cn/.

[53] Institute of Botany, Chinese Academy of Sciences (1996). China Vegetation Database.

[54] Hurlbert AH, White EP (2005). Disparity between range map- and survey-based analyses of species richness patterns, processes and implications.. Ecology Letters.

[55] Hurlbert AH, Jetz W (2007). Species richness, hotspots, and the scale dependence of range maps in ecology and conservation.. Proceedings of the National Academy of Sciences USA.

[56] Luo Z, Tang S, Li C, Chen J, Fang H, Jiang Z (2011). Do Rapoport's rule, mid-domain effect or environmental factors predict latitudinal range size patterns of terrestrial mammals in China?. PLoS ONE.

[57] Burnham KP, Anderson DR (2002). Model selection and multimodal inference: a practical information-theoretic approach, second edition.

[58] Murray K, Conner MM (2009). Methods to quantify variable importance: implications for the analysis of noisy ecological data.. Ecology.

[59] Diniz-Filho JAF, Bini LM, Hawkins BA (2003). Spatial autocorrelation and red herrings in geographical ecology.. Global Ecology and Biogeography.

[60] Wright DH, Currie DJ, Maurer BA, Ricklefs RE, Schluter D (1993). Energy supply and patterns of species richness on local and regional scales.. Species diversity in ecological communities: historical and geographical perspectives.

[61] Brown JH (1981). Two decades of homage to Santa Rosalia: toward a general theory of diversity.. Integrative and Comparative Biology.

[62] Rosa R, Dierssen HM, Gonzalez L, Seibel BA (2008). Ecological biogeography of *cephalopod molluscs* in the Atlantic Ocean: historical and contemporary causes of coastal diversity patterns.. Global Ecology and Biogeography.

[63] Adams JM, Woodward FI (1989). Patterns in tree species richness as a test of the glacial extinction hypothesis.. Nature.

[64] Cousins SH (1989). Species richness and the energy theory.. Nature.

[65] Guegan JF, Lek S, Oberdorff T (1998). Energy availability and habitat heterogeneity predict global riverine fish diversity.. Nature.

[66] Hurlbert AH (2004). Species-energy relationships and habitat complexity in bird communities.. Ecology Letters.

[67] Storch D, Davies RG, Zajicek S, Orme CDL, Olson V (2006). Energy, range dynamics and global species richness patterns: reconciling mid-domain effects and environmental determinants of avian diversity.. Ecology Letters.

[68] Whittaker RJ, Nogués-Bravo D, Araújo MB (2007). Geographic gradients of species richness: a test of the waterenergy conjecture of Hawkins *et al*. (2003) using European data for five taxa.. Global Ecology and Biogeography.

[69] Schall J, Pianka E (1978). Geographical trends in numbers of species.. Science.

[70] Turner JRG, Lennon JJ, Lawrenson JA (1988). British bird species distributions and the energy theory.. Nature.

[71] Hawkins BA, Montoya D, Rodríguez M, Olalla-Tárraga M, Zavala M (2007). Global models for predicting woody plant richness from climate: comment.. Ecology.

[72] Qian H, Badgley C, Fox DL (2009). The latitudinal gradient of beta diversity in relation to climate and topography for mammals in North America.. Global Ecology and Biogeography.

[73] Altamirano A, Field R, Cayuela L, Aplin P, Lara A (2010). Woody species diversity in temperate Andean forests: The need for new conservation strategies.. Biological Conservation.

[74] Tilman D, Fargione J, Wolff B, D'Antonio C, Dobson A (2001). Forecasting agriculturally driven global environmental change.. Science.

[75] Sang A, Teder T, Helm A, Pärtel M (2010). Indirect evidence for an extinction debt of grassland butterflies half century after habitat loss.. Biological Conservation.

[76] MacArthur RH, Wilson EO (1967). The theory of island biogeography.

[77] MacArthur RH, MacArthur JW (1961). On bird species diversity.. Ecology.

[78] Carroll C (2010). Role of climatic niche models in focal-species-based conservation planning: Assessing potential effects of climate change on Northern Spotted Owl in the Pacific Northwest, USA.. Biological Conservation.

